# Review of evidence based clinical practice guidelines developed in Latin America and Caribbean during the last decade: an analysis of the methods for grading quality of evidence and topic prioritization

**DOI:** 10.1186/s12992-019-0455-0

**Published:** 2019-02-19

**Authors:** Paula Andrea Cabrera, Rodrigo Pardo

**Affiliations:** 1 Faculty of Medicine, Universidad Nacional de Colombia, Bogotá D.C., Colombia; 2 Clinical Research Institute and Health Technology Assessment Unit, Faculty of Medicine, Universidad Nacional de Colombia, Bogotá D.C., Colombia

**Keywords:** Caribbean region, GRADE, Latin America, Practice guideline

## Abstract

**Background:**

In the last decade, efforts have been made in Latin America and the Caribbean to advance in the methodological development of evidence based clinical practice guidelines, among other strategies to improve the health provision of services and indicators.

**Objectives:**

To build an evidence map to show the regional GRADE impact in developing clinical practice guidelines and contrast the results with current needs.

**Methods:**

A systematic literature search was conducted in databases, developer’s websites, health ministries, repositories and grey literature. Documents were included when they were evidence based clinical practice guidelines developed in Latin American and Caribbean countries in the last decade. Data from the *Global Burden of Disease* was used to highlight relevant health conditions.

**Results:**

Nine thousand seven hundred seventy-six documents were retrieved. 98 guidelines, with specific mention of the use of GRADE methodology were identified. 81% of the guidelines were developed within the last 4 years. 68% are from Colombia, 13% from Peru, 9% from Chile, 3% from Argentina and Costa Rica and Brazil, Honduras and Dominican Republic account 1%. 67% were developed for non-communicable diseases, 10% for communicable diseases, 9% for neonatal pathologies and 5% for maternal problems, 1% injuries and 7% other topics (nutrition, oral health).

**Discussion:**

Our findings show a slow and increasing incorporation of the GRADE methodology in the region. GRADE guidelines have been adopted mainly by Colombia and slowly by other countries. Topics for guidelines continue to be comparable to the high-income countries and they don’t address communicable diseases or other relevant health issues in the region, such as violence or malnutrition; thus, the evidence based guidelines for clinical practice are only a tool within a complex multimodal strategy to tackle the challenges of the health determinants.

**Conclusions:**

A prioritizing strategy for relevant regional health topics and the use of robust methodological approaches must be in the political agenda in the region. GRADE methods could help to improve the quality and validity of recommendations not only for chronic pathologies but also for ancient and challenging maladies prevalent in the region, as part of a multimodalintersectoral strategy.

**Electronic supplementary material:**

The online version of this article (10.1186/s12992-019-0455-0) contains supplementary material, which is available to authorized users.

## Background

Evidence based clinical practice guidelines (CPG) are an efficient strategy to optimize health care through the implementation of valid recommendations for specific conditions. They guide health professionals and decision makers in areas of clinical uncertainty [1–4] through evidence-based recommendations that assess the benefits-risk balance and critically appraise old and new technologies.

CPG are documents developed with methodological rigour by multidisciplinary panels that incorporate, not only valid results of ongoing research, but also the opinion and experience of clinicians, patient’s preferences and values, priorities and needs within the community, available resources and costs, legal frameworks, cultural heterogeneity and health system organization [1, 5–7]. CPG must be valid and replicable, multidisciplinary and collaborative, easily applicable, accessible, unambiguous, having the aim to increase their reliability, acceptance, use and implementation. They are the result of an active and planned process that takes into consideration the barriers and facilitators to implement the recommendations in the daily clinical scenarios for the local, regional or national contexts they are designed for [4, 5, 7].

Leading countries have a solid infrastructure dedicated to the production of high-quality CPG from the best available evidence, which become national and international benchmarks. In Latin America and the Caribbean, efforts have been made to further the methodological constructions of these documents [1, 5].

National programs have been created in the region to support the systematic development of guidelines. Initiatives such as the ones from National Academy of Medicine of Argentina (2006), the Brazilian Medical Association, the Brazilian Ministry of Health (2004), the AUGE initiative (Chile 2005), the Ministry of Health and Social Protection from Colombia (2009), the IHCAI Foundation of Costa Rica (2004), or the National Center for Technological Excellence in Health of the Mexican government (2007) are worth mentioning, among new emerging ones.

This ongoing process has been made possible thanks to the commitment of relevant partners from Spain (Guiasalud, Enebro Foundation and the Universidad de Sevilla), Portugal (Centro de Estudos de Medicina Baseada na Evidência, Faculdade de Medicina da Universidade de Lisboa), the Pan American Health Organization (PAHO) and from the Guidelines International Network (GIN) that harmonize and systematize the development of CPG around the world.

To our knowledge, no systematic evaluation has been done during the last decade to assess the development of CPG and the incorporation of new methodologies in the Latin American and Caribbean region.

This paper presents a mapping of regional evidence CPG that recognizes the commitment of the Latin American and Caribbean countries, as well as a critical view of how the CPG and the methodologies for quality evidence grading have managed to insert themselves in the different countries to provide reliable evidence-based recommendation for the regional burden of morbimortality and which have an effect on public policy.

## Materials and methods

We made a systematic search of the literature to describe cross-sectional CPGs developed in Latin America and the Caribbean in the last 10 years.

### Study selection

The documents were included when: (i) they were CPGs with explicit recommendations and evidence based grading system, (ii) they focus on patient care level (iii) developed in Latin American Countries and (iv) endorsed by a government agency or the corresponding entity for national use, without restriction of language or methodology employed.

Documents were excluded when: (i) they were classified as standard, routine care manual or a protocol due to their methodological characteristics; (ii) they were CPGs based on expert consensus, without a methodological systematic approach; (iii) they addressed patients or stakeholders using a public health approach (iv) when the complete version was not available, or, (v) if the year or scope could not be established. The relevant languages for the area (Spanish, English, French and Portuguese) were included.

The research strategy was restricted on clinical guidelines as the main focus. The decision to exclude public health guidelines was based on the fact that quality assessment methods have been developed selectively for clinical problems and the quality assessment tools in public health guidelines may require a different approach. These limitation may be overcome as the guidelines on public health issues move forward to high quality standards.

### Search strategy

Electronic search was conducted on MEDLINE, Scientific Electronic Library Online (SciELO) and Embase databases.

An internet search of developer’s websites, health ministries, electronic practice guidelines repositories, and generic search engines were also included as proposed by the Methodological Guide for the Development of Guidelines in the General Security Health System of Colombia and in the Guidelines for the strengthening of national programs of evidence based guidelines from the World Health Organization [4, 5].

The aim of the research was to identify relevant documents using the following keywords: CPG, management guide, care guide. The terms protocol, standard, routine care manual, and recommendation were also included to acknowledge nomenclature diversity in the region and to improve the search sensitivity.

### Data extraction, synthesis and assessment

The complete text evidence-based CPGs included was then classified according to the evidence grading methodology employed: Oxford, Scottish Intercollegiate Guidelines Network (SIGN), Grading of Recommendations Assessment, Development and Evaluation (GRADE), other methodologies and others which did not define a specific methodology.

Analysis was restricted to CPGs that incorporate the GRADE methodology as established by its developer group. Methodological rigour and transparency in the production of documents were quantified using the GRADE methodology, since it implements a unified and systematic approach that determines the strength and direction of recommendations [8].

GRADE is currently considered the best methodology to build valid and transparent recommendations given its strict evaluation of bias in the available information, the magnitude and stability of the effects, the presence of confounding factors, inconsistencies or other quality issues [9]. Recommendations are formulated considering not only the quality of the evidence, but the risk-benefit balance, the preferences of the patients, and the costs, ensuring high-quality guidelines for successful adaptation processes [9].

The information obtained after conducting the search and selecting the documents was compared with the regional morbimortality indicators as presented by the Global Burden of Disease initiative (GBD) to determine how the initiatives correlate with the actual health needs and challenges. Descriptive statistical analysis was done through Stata [10].

## Results

### Regional guideline production and methodological implementation

**Fig. 1:**
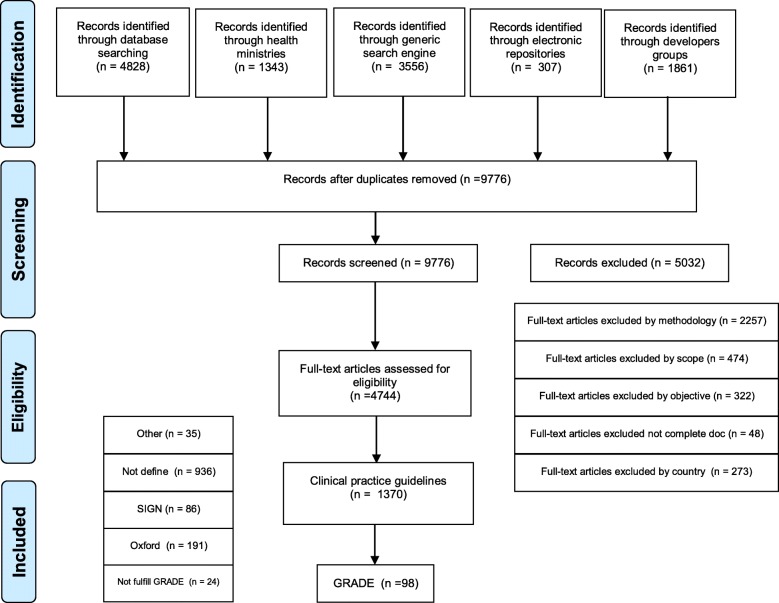
Flow diagram. Documents identified, screened and selected

A total of 9776 documents were screened by title and abstract using the search methodology. 4744 articles were then selected to assess inclusion and exclusion criteria. Excluded documents were classified as follows: i) protocols, routine care manuals or standards (*n* = 2257) due to their lack of methodological rigour for an evidence based CPG, ii) not having a nation-wide scope developed by scientific societies or non-governmental institutions (*n* = 474), iii) addressing public health issues (vaccination programs or vector control programs) and macro decisions, or that were directed to stakeholders or patients (*n* = 322); and iv) documents excluded either because they were related to countries not in the region or because their complete text could not be retrieved (*n* = 321) (Fig. 1).

One thousand three hundred seventy documents fulfilled the inclusion criteria. They were then classified according to the methodology used to assess the quality of the evidence as follows: i) 936 CPGs that defined their own assessment scale or didn’t state the specific methodology used; ii) 191 declared using Oxford; iii) 86 used SIGN; iv) 35 used other specified methodologies; and v) 122 used the GRADE methodology.

**Fig. 2:**
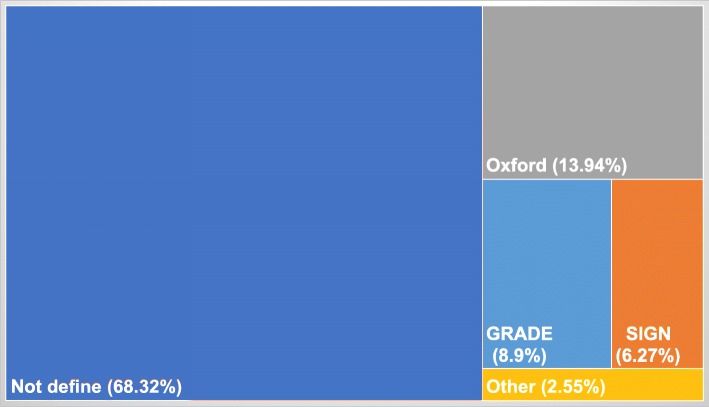
Methodological evidence quality assessment distribution. GRADE: Grading of Recommendations Assessment, Development and Evaluation; SIGN: Scottish Intercollegiate Guidelines Network

From the 122 documents that fulfilled the inclusion criteria, we analysed 98 guidelines which met the GRADE methodology. Figure 2 represents the distribution (%) of clinical guidelines grading methodology across the region. An additional dataset file shows this in more detail (see Additional file 1).

**Fig. 3:**
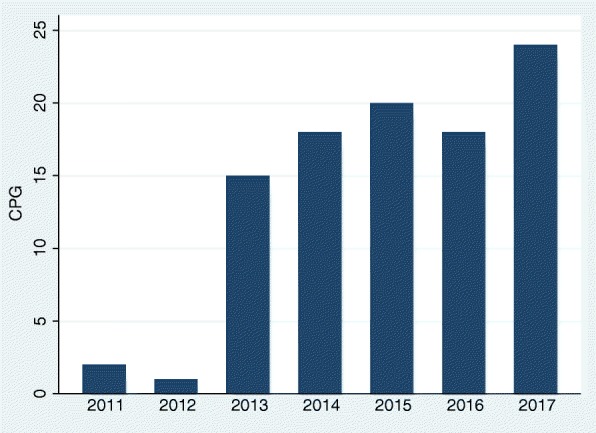
Temporal evolution of GRADE methodology incorporation. CPG: clinical practice guidelines

From a historical perspective, the incorporation of the GRADE methodology started in 2011 and increased over time, with 81% of the documents being developed within the last 4 years (Fig. 3).

**Fig. 4:**
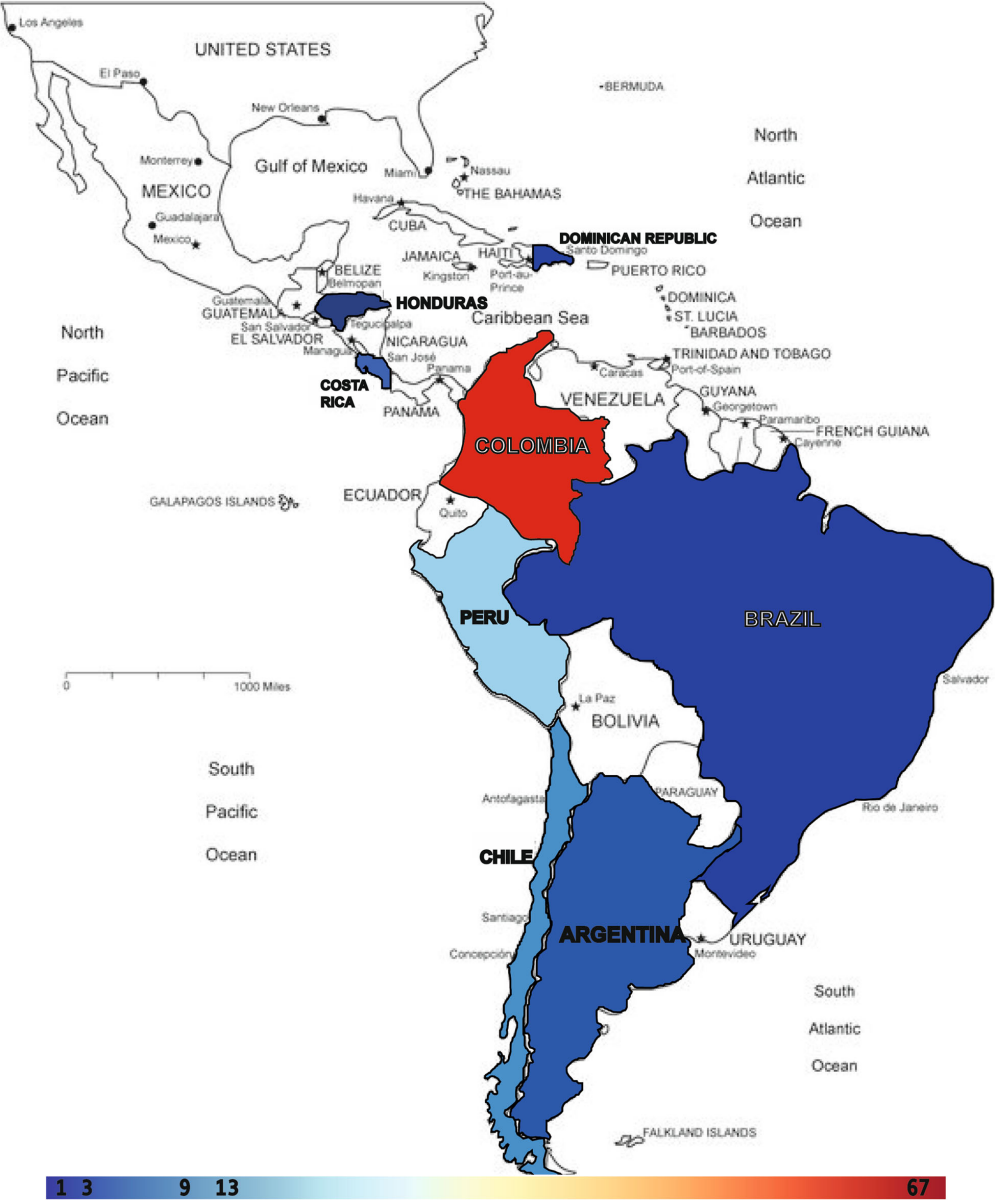
Geographical distribution of GRADE implementation. Color scale represents absolute number of GRADE CPG production by country. GRADE: Grading of Recommendations Assessment, Development and Evaluation

68% of documents were from Colombia. The rest came from: Peru (13%), Chile (9%), Argentina (3%), Costa Rica (3%), and Brazil, Honduras and Dominican Republic with 1% each. Figure 4 shows the geographical density distribution after mapping GRADE’s implementation.

Developers were classified according to the levels defined by Esandi et colleagues as follows: i) macro if they were developed by the national entity in charge of public policy formulation and control, such as health ministries, ii) meso when developed by intermediate institutions that manage health services; and iii) micro when developed by as scientific or professional health associations. According to Table 1, the health ministries were the main CPG developers (67/98), followed by the meso (16/98) and micro level (15/98) institutions. An interaction between levels was observed, where the meso and micro levels cooperated actively with the macro level [11].

**Table 1:** CPG discriminated by health system level production

Health system level	CPG guidelines	Percent	Cum.
Macro	67	68.37	68.37
Meso	16	16.33	84.69
Micro	15	15.31	100.00
Total	98	100.00	

### Non-methodological CPG assessment

In the selected CPGs, aspects not related to the methodology but relevant to the whole process were considered (Table 2): 98% CPGs declared conflict of interest and editorial independence, while 85% declared funding. 59% of the CPG’s included an implementation plan, and 46% included an economic analysis.

**Table 2:** Non methodological CPG assessment

	CPG guidelines	Percent
Declaration of conflict of interest and editorial independence	97	98.98
Declared funding	84	85.71
Implementation plan	58	59.18
Economic analysis	46	46.94

### Topic prioritization

**Fig. 5:**
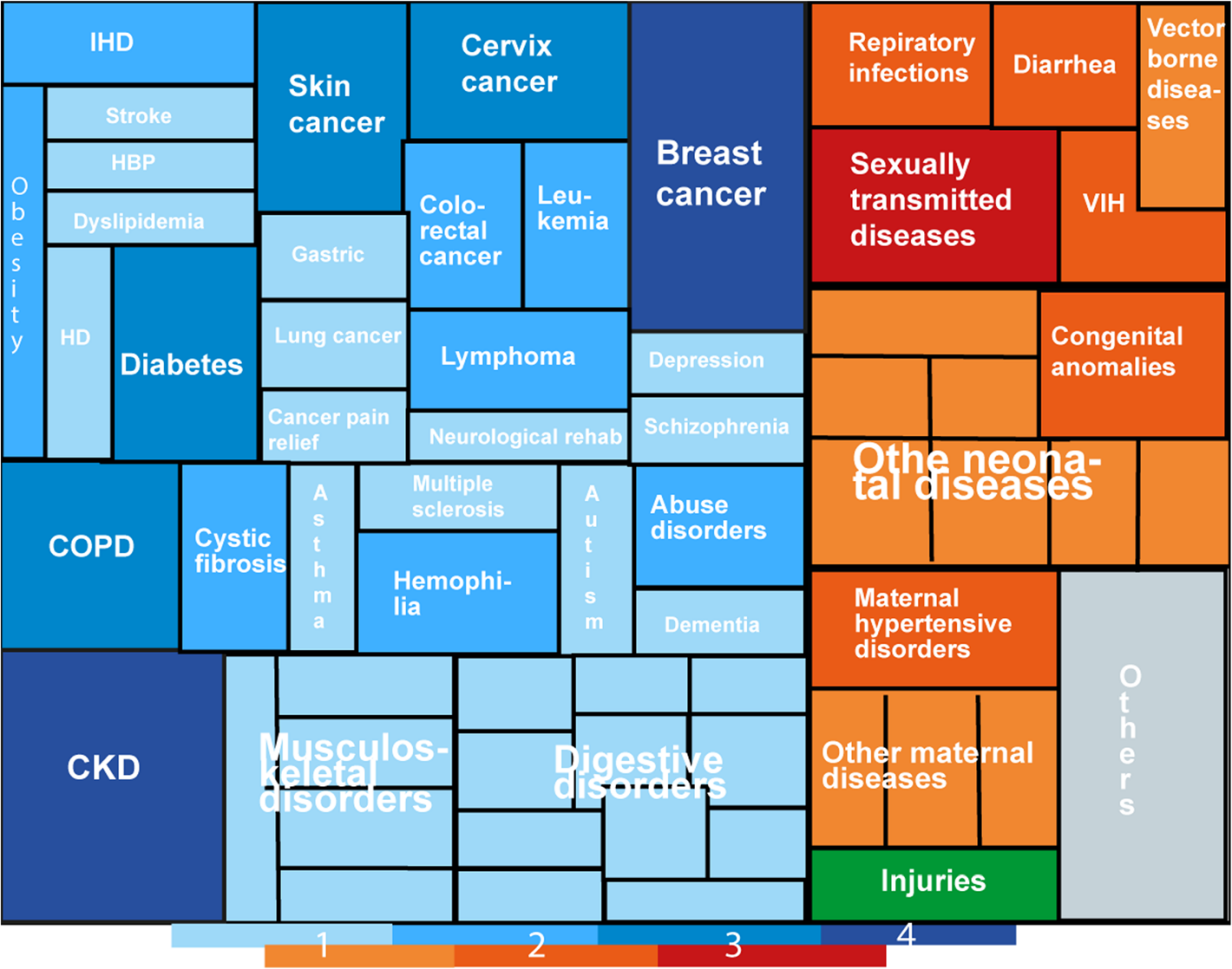
Percentage distribution of CGP topic prioritization. Colors represent three main morbimortality groups as follows: i) Blue: Non-communicable diseases, ii) Red: communicable, neonatal, maternal and nutritional diseases, iii) Green: injuries. Sub classification accounts for specific pathologies, with color scale and size representing total number of CPG by specific pathology. This diagram has been designed following GBD strategy in order to highlight priorities. CKD: Chronic Kidney Disease; COPD: Chronic Pulmonary Disease; HBP: High Blood Pressure; HD: Heart Disease; IHD: Ischemic Heart Disease

67% of the topics pertain to non-communicable diseases, where neoplasm and digestive pathologies (Table 3) are significant. Communicable (10%), neonatal (9%), maternal (5%) diseases, injuries (1%) and others (7%) that include nutrition, anaesthesia and dental health account for the remaining topics as shown in Table 4. Figure 5 represents the graphical distribution (%) of CPGs topic prioritization in Latin America and the Caribbean, classified into three main groups according to the causes of morbimortality as defined by the GBD initiative, and then sub classified by specific pathologies [10].

**Table 3:** Percentual distribution of non-communicable diseases CPGs

Pathology	Freq.	Percent	Cum.
Neoplasms	19	28.79	28.79
Digestive	11	16.67	45.45
Mental and behavioral	6	9.09	54.55
Respiratory	6	9.09	63.64
Metabolic Cardiovascular	6	9.09	72.73
Cardiovascular	5	7.58	80.30
Musculoskeletal	5	7.58	87.88
Renal-urinary	4	6.06	93.94
Hematologic	2	3.03	96.97
Neurologic	2	3.03	100.00
Total	66	100.00

**Table 4:** Percentual distribution of CGP topic prioritization by main groups

Group	CPG	Percent	Cum.
Non-communicable diseases	66	67.35	67. 35
Infectious - communicable diseases	10	10.20	77.55
Neonatal diseases	9	9.18	86.73
Others	7	7.14	93.88
Maternal diseases	5	5.10	98.98
Injuries	1	1.02	100.00

71% of the CPGs addressed diagnosis and 93% treatment/management, while 30% were directed to prevention or screening practices, and 12% were related to follow-ups or palliative care (Table 5).

**Table 5:** Address clinical aspect by CPG

Clinical aspect	CPG	Percent
Screening	13	13.27
Prevention	17	17.35
Diagnosis	70	71.43
Treatment	92	93.88
Follow-up	19	19.39
Palliative care	2	2.04
Rehabilitation	10	10.20

### Global burden of disease: Latin America and Caribbean

Latin America and the Caribbean countries are experiencing demographic changes, due to population growth and aging. There has been an increased burden of non-communicable diseases, such as ischemic heart disease, mental disorders, musculoskeletal diseases, diabetes, cirrhosis, and chronic kidney disease that follow global trends. Other important causes of disability in the region include road accident related injuries, drug and alcohol use related disorders and personal violence [10].

Premature deaths due to poverty related communicable diseases, or from neonatal, nutritional and maternal causes have decreased in the region [10].

Even though the above-mentioned statements are true, there are interregional differences. In low and lower middle-income countries such as Bolivia, Guatemala, Guyana and Haiti the burden of communicable diseases is high. HIV remains a challenge to address as it continues to be an important cause of death in the region. By contrast, the morbimortality rates in upper middle-income countries are comparable to global trends [10].

## Discussion

During the last decade Latin American and Caribbean countries have recognized CPG as an important strategic methodology to incorporate quality, timeliness, equality, efficient use of resources and safety in healthcare, as shown by the increasing amount of evidence based CPGs documents, yet there have been historical difficulties in the construction of recommendations: poor methodological rigour, a non-systematic approach and inaccurate strategy to search for, select or grade evidence [2].

In some other cases, because of the remarkable heterogeneity in the writing process or format presentation it is difficult for the user to identify the recommendations, or to link them to the supporting evidence, patient preferences or costs.

Therefore, regional CPGs are susceptible to bias, raising concern about their quality, validity and reliability. As reported by several authors who specifically address this issue, the weaknesses of the CPGs in Latin America and the Caribbean include: lack of transparency, rigour, or methodological objectivity; absence of multidisciplinary work teams; non-inclusion of patient preferences; inadequate descriptions of evidence searches and selection of grading methods employed; inaccurate formulation of recommendations; lack of specific objectives, scope or target populations; low stakeholder involvement; and insufficient implementation tools [11–20].

Our results show a slow and progressive incorporation of high-quality methodologies. GRADE, as a small fraction of the evidence based CPG, is the result of an initial regional push, where the method has managed to overcome multiple barriers such as the health systems complexity, absence of an established methodological systematic approach, lower consolidation of evidence based medicine, language barriers to access medical research literature, lack of cumulative experience and unavailable economic and human resources to accomplish reliable and high quality outcomes in the region [11, 12].

Colombia has established a national program for the development of CPGs as a governmental response to improve healthcare quality and equality. Under a strategy of knowledge transfer, the National Institute for Clinical Excellence (NICE) and GIN endorsed the systematic development of high-quality GRADE evidence-based CPGs balancing interest, knowledge, expectations, opinions, and preferences to formulate valid recommendations [2].

Like Colombia, other Latin American countries are currently running GRADE methods to build high quality recommendations. CPGs from Chile, Peru, Argentina, Costa Rica and Dominican Republic have gradually incorporated the GRADE methodology under the leadership, coordination and supervision of governments and health ministries, with the active involvement of institutes, scientific societies, universities and research groups, as a multidisciplinary and participative approach.

Secondary indicators assessing non-methodological issues such as the reporting of Statements of conflict of interest and funding levels were high among GRADE CPGs, while the plans for their implementation and their economic analysis were reported in half of them.

Implementing robust methodologies will enable our countries to build a regional collaborative framework to develop CPG guidelines with the highest standards that sustain a feasible adaptation process, taking advantage of available knowledge and avoiding the duplication of efforts. This may allow the formulation of tailor-made recommendations to improve transferability in our context, reducing costs and shortening the gap between evidence and daily clinical practice [5].

Additionally, the potential of CPGs to bring medical breakthroughs into health policies and clinical outcome improvements implicitly includes the needs of the population. As shown in our results, regional CPGs topic prioritization is comparable to the high-income countries (HIC) as reported by Global Burden of Disease initiative [10].

Epidemiological transition occurs unequally, and traditional poverty related diseases that affect our countries are not being properly addressed and persist in low and lower middle income countries [10]. There is a lack of regional initiatives for the formulation of evidence recommendations that address specific pathologies such as malaria, leprosy, Chagas, tuberculosis and other millennial evils [6, 7].

Clinical aspects covered by CPG focus mainly of diagnosis and treatment, neglecting prevention and promotion efforts that would effectively minimize the disease burden. These may be partially explained by the fact that, in contrast with public health guidelines, CPG do no necessary address issues as dietary risk factors, high body mass index, high blood pressure, high fasting glucose and alcohol use as significant causes of premature death and disability in the region [10].

Given the high burden of unsatisfied health needs, prioritization of topics using multilevel criteria becomes crucial in the process of allocating resources in Latin America and the Caribbean to select which CPGs to develop according to economic implications, impact on health system, social outcomes, feasibility, and effectiveness [21].

Within a complex multimodal health strategy, CPGs represent a strategy to tackle the challenges of the health determinants in national programs that should articulate with other necessary and important initiatives from the public health, economic, and social sectors to improve health and equity in Latin America and Caribbean countries.

Regionally, health determinants are not fully satisfied and there are persistent wide socioeconomic inequalities with one of the highest Gini index among the world as a representation of inequitable distribution of resources and power. Treating diseases that share the fearsome attribute of resistance while they further impoverish three quarters of the world’s population living in extreme poverty is incredibly challenging, and requires shared efforts from the clinical and the public health fields.

Achieving sustainable development involves multisectoral articulated initiatives that approach social determinants with redistributive policies reforms. Extreme poverty, rapid population growth, migratory pressures, political disputes, and mediocre sanitary conditions must be forcefully addressed with the goal to have an inclusive and equitable society [22].

Evidence based medicine is a reasonable strategy to efficiently acknowledge health issues, reducing costs with interventions that do work, which becomes important where scarcity of resources is the rule. However, whether EBM and CPG themselves guarantee optimal care for the patient is an open debate and to what extent they impact health indicators, especially in Latin America and the Caribbean countries is highly questionable [23].

This is arguable since EBM serves the best interest of the pharmaceutical industry, as they decide research agendas, establish surrogate endpoints for efficacy and overestimate marginal benefits in a saturated therapeutic field. It is considered by some authors a normative regimen of truth, as it is stated the only valid and truthful method for knowledge generation, while dismissing and eliminating other methods considered imperfect. It denies creativity and plurality that may come to play important role in a reflective process that considers ethical and existential issues of patient care [23, 24].

Clinical evidence is mainly produced in developed countries and sophisticated research centres where major health concerns differ. Important health issues in developing countries have not yet been reviewed because of lack of scientific funding for high quality research. Interventions proposed by EBM are usually high cost technologies not available in the region, where clinicians rely on old, cheaper technologies, while evidence from randomized controlled trials may not be transferable in the setting of developing countries [25].

Feasible disease interventions and recommendations based on Latin American specific challenges and resources must be formulated to overcome implementation barriers and be accepted by physicians as a synergistic action along with other initiatives that address social determinants.

Finally, some methodological limitations of this review must be addressed. Although a systematic approach was intended, search and selection strategy was done unpaired and restricted to their web availability, as then could exclude CPGs that may have not been sensitive to the established search strategy. Secondly, the search focus was mainly on guidelines with a clinical scope, and public health guidelines were excluded even when recognizing them as essential element to address social determinants in the region, as they mainly remark prevention and promotion efforts, not uninformedly included in CPG. Thirdly, analysis and assessment was done only on GRADE methodology as first approximation, excluding other relevant CPGs initiatives.

In the future a more comprehensive analysis must be considered, as further including other methodological approaches and public health initiatives will enable a better understanding of current situation of practice guidelines in the region.

## Conclusion

This approach recognizes efforts of Latin American and Caribbean countries in the production of CPGs and the incorporation of the methods for grading quality of evidence and topic prioritization. In accordance to our results, although regional CPG production has markedly increased over time, their incorporation has been slow; mainly due to the lack of a definition in national policies agendas that systematizes methodological rigour.

Continuous efforts must be made to introduce methodological improvements to generate CPGs in Latin America and the Caribbean. A regional unified collaborative framework must be adopted to avoid duplication, improve efficacy and meet the morbimortality particularities of the region, especially for poverty related diseases. We recognize this as an initial approach, more epidemiological research is needed to broaden regional GPG development knowledge.

EBM limitations must be recognized while it is still open debate whether EBM along CPG by themselves may directly influence health indicators, as health processes within Latin American and Caribbean countries are the result of highly complex interactions of key social determinants.

## Additional file

Additional file 1: GRADE clinical practice guidelines. Data extraction of regional GRADE clinical practice guidelines. (XLSX 55 kb)

## Abbreviations

CPG: Clinical practice guidelines
EBM: Evidence based medicine
GIN: Guideline International Network
GRADE: Grading of Recommendations Assessment, Development and Evaluation
HIC: High income countries
NICE: National Institute for Clinical Excellence
PAHO: Pan American Health Organization
SciELO: Scientific Electronic Library Online
SIGN: Scottish Intercollegiate Guidelines Network
